# Identifying drug targets for neurological and psychiatric disease via genetics and the brain transcriptome

**DOI:** 10.1371/journal.pgen.1009224

**Published:** 2021-01-08

**Authors:** Denis A. Baird, Jimmy Z. Liu, Jie Zheng, Solveig K. Sieberts, Thanneer Perumal, Benjamin Elsworth, Tom G. Richardson, Chia-Yen Chen, Minerva M. Carrasquillo, Mariet Allen, Joseph S. Reddy, Philip L. De Jager, Nilufer Ertekin-Taner, Lara M. Mangravite, Ben Logsdon, Karol Estrada, Philip C. Haycock, Gibran Hemani, Heiko Runz, George Davey Smith, Tom R. Gaunt, AMP-AD eQTL working group

**Affiliations:** 1 MRC Integrative Epidemiology Unit (IEU), Population Health Sciences, University of Bristol, Bristol, United Kingdom; 2 Translational Biology, Research and Development, Cambridge, Massachusetts, United States of America; 3 Sage Bionetworks, Seattle, Washington, United States of America; 4 Department of Neuroscience, Mayo Clinic Florida, Jacksonville, Florida, United States of America; 5 Department of Health Sciences Research, Mayo Clinic Florida, Jacksonville, Florida, United States of America; 6 Centre for Translational & Computational Neuroimmunology, Department of Neurology, Columbia University Medical Centre, New York, New York, United States of America; 7 Taub Institute for Research on Alzheimer’s Disease and the Aging Brain, Columbia University Irving Medical Centre, New York, New York, United States of America; 8 Department of Neurology, Mayo Clinic Florida, Jacksonville, Florida, United States of America; 9 BioMarin Pharmaceuticals, San Rafael, California, United States of America; 10 NIHR Bristol Biomedical Research Centre, Oakfield House, University of Bristol, Bristol, United Kingdom; University of Miami, Miller School of Medicine, UNITED STATES

## Abstract

Discovering drugs that efficiently treat brain diseases has been challenging. Genetic variants that modulate the expression of potential drug targets can be utilized to assess the efficacy of therapeutic interventions. We therefore employed Mendelian Randomization (MR) on gene expression measured in brain tissue to identify drug targets involved in neurological and psychiatric diseases. We conducted a two-sample MR using cis-acting brain-derived expression quantitative trait loci (eQTLs) from the Accelerating Medicines Partnership for Alzheimer’s Disease consortium (AMP-AD) and the CommonMind Consortium (CMC) meta-analysis study (n = 1,286) as genetic instruments to predict the effects of 7,137 genes on 12 neurological and psychiatric disorders. We conducted Bayesian colocalization analysis on the top MR findings (using P<6x10^-7^ as evidence threshold, Bonferroni-corrected for 80,557 MR tests) to confirm sharing of the same causal variants between gene expression and trait in each genomic region. We then intersected the colocalized genes with known monogenic disease genes recorded in Online Mendelian Inheritance in Man (OMIM) and with genes annotated as drug targets in the Open Targets platform to identify promising drug targets. 80 eQTLs showed MR evidence of a causal effect, from which we prioritised 47 genes based on colocalization with the trait. We causally linked the expression of 23 genes with schizophrenia and a single gene each with anorexia, bipolar disorder and major depressive disorder within the psychiatric diseases and 9 genes with Alzheimer’s disease, 6 genes with Parkinson’s disease, 4 genes with multiple sclerosis and two genes with amyotrophic lateral sclerosis within the neurological diseases we tested. From these we identified five genes (*ACE*, *GPNMB*, *KCNQ5*, *RERE* and *SUOX*) as attractive drug targets that may warrant follow-up in functional studies and clinical trials, demonstrating the value of this study design for discovering drug targets in neuropsychiatric diseases.

## Author summary

Genetic association studies have been successful in identifying many genetic variants associated with disease risk, but it has been far more challenging to determine the genes through which these act. This is important, because such genes may encode effective drug targets for these diseases. We used Mendelian randomization (MR) and colocalization, two methods which in combination exploit these genetic variants to estimate the causal effects of individual genes. We applied this approach to 12 neurological and psychiatric diseases using data from the AMP-AD and CMC brain expression quantitative locus dataset, which is large enough to provide robust evidence for the relationship between genetic variants and gene expression. We found a causal relationship between the change in expression of 47 genes and increased disease risk across the 12 diseases we tested. As drug targets with human genetic evidence are far more likely to be approved in clinical trials, these findings provide a valuable list of potential therapeutic targets, including the *ACE*, *GPNMB*, *KCNQ5*, *RERE* and *SUOX* genes.

## Introduction

There is a compelling need to improve the processes to discover new drugs for human disease, which is time consuming and expensive, with high rates of attrition [1]. Attrition rates have been particularly high for therapies against brain-related diseases. For instance, a survey of 413 Alzheimer’s disease trials reported a failure rate of 99.6% [2]. Neurological and psychiatric diseases are highly heritable [3]. Genome-wide association studies (GWAS) have identified numerous genetic loci associated with disease risk [4,5], and can therefore provide evidence to identify molecular pathways involved in disease that can be intervened on by drugs. Indeed, drug targets with human genetic evidence are more than twice as likely to be approved than those without [6,7].

One approach to nominate potential drug targets through genetics is to integrate GWAS associations with gene expression quantitative trait loci (eQTLs) to select the most likely functional variants and the genes controlled by these variants. Recent studies have adopted such an approach through a combination of two sample Mendelian randomization (MR) and colocalization methods to infer whether a causal relationship exists between gene expression and disease outcomes. For example, two sample MR studies have systematically explored causal relationships between the transcriptome [8,9,10]. methylome [11] and proteome [12] and complex traits. Zhu et al performed MR between blood eQTLs and five complex traits and found potential causal effects for 126 genes in total, including 25 novel genes previously unreported in the literature [9]. Richardson et al [11] and Zheng et al [12] performed MR on a wider phenome-wide scale and found putative causal effects for 1,148 methylation quantitative trait loci (mQTLs) on 139 complex traits and 111 protein quantitative trait loci (pQTLs) on 225 complex traits respectively. After causal variants have been identified, a follow up MR phenome-wide association analysis [13] can then be conducted to assess a gene’s suitability as a drug target. Specifically, it can be assessed whether associations to clinical endpoints match the intended indications, or whether there might be a risk for adverse events [14].

A major challenge for two sample MR of gene expression with brain diseases is to obtain tissue-specific expression datasets at large enough sample sizes to be powered for obtaining robust eQTL associations to instrument genes. In this study, we utilized summary statistics from a recent cerebral cortex eQTL meta-analysis (n = 1,286) that combined RNA-seq data from the Accelerating Medicines Partnership for Alzheimer’s Disease consortium (AMP-AD) and the CommonMind Consortium (CMC) [15] (henceforth referred to as AMP-CMC). We extracted 7,689 eQTL instruments from this study and conducted a transcriptome-wide two sample MR and colocalization study between brain eQTLs and 12 neurological and psychiatric conditions. By identifying shared genetic effects between brain-specific gene expression and Central Nervous System (CNS) disease, our study establishes causal roles for several genes across the conditions tested that could be starting points for the development of efficient drugs.

## Results

### Selection of instrumental variables for Mendelian Randomization from AMP-CMC

AMP-CMC combines RNA-seq data from 1,286 human cerebral cortex samples from the Accelerating Medicines Partnership for Alzheimer’s Disease (AMP-AD, composed of the ROSMAP and MAYO cohorts) and the CommonMind consortia (CMC, composed of the MSSM-Penn_Pitt cohorts) [15]. We first assessed how eQTLs used as instruments from this cohort compared to alternative brain eQTL datasets available for public download, specifically a meta-analysis across AMP, CMC and GTEx on pre-frontal cortex tissue (brain cortex meta-analysis), and a meta-analysis across 10 different brain tissues available in GTEx (GTEx brain meta-analysis) [10]. Gene expression effects for these brain eQTL datasets agreed well with our study (r = 0.72, 95% CI [0.71,0.73] brain cortex meta-analysis, r = 0.70, 95% CI [0.68, 0.71] for GTEx brain meta-analysis). Of the 6,790 harmonised brain cortex meta-analysis effects, 3,922 (57.8%) effects achieved a genome-wide significance level used to select instruments in this study (P<5x10^-8^). An additional 2,224 effects (32.8%) reached nominal significance (P<0.05) and 644 effects (9.5%) showed no evidence of association (P>0.05). Of the 6,628 harmonised effects from the GTEx brain meta-analysis, 1,642 (24.8%) effects reached P<5x10^-8^, an additional 3,256 (49.1%) reached P<0.05, and 1,730 (26.1%) showed no evidence of association (P>0.05). Of the nominally significant effects, only 49 (0.8%) and 71 (1.4%), respectively, showed opposite directionality of effect relative to our study (S1 Table).

Blood eQTLs are sometimes used as instruments when examining brain-related disorders, but this approach can be problematic for genes that are differentially expressed between brain and blood. To better understand differences, we compared AMP-CMC instruments against the currently largest public blood eQTL resource, eQTLGen [16]. As expected, the correlation between blood and brain across all gene expression effects was modest (r = 0.48, 95% CI [0.46,0.50]). However, of the 5,820 harmonised eQTLGen effects, 4,133 (71.0%) effects reached P<5x10^-8^ and an additional 984 effects (16.9%) nominal significance (P<0.05). Interestingly, a substantial proportion of eQTLs (1,028 or 21.2% of the 4,836 eQTLGen effects at P<0.05) showed opposite direction of gene expression effect between blood and brain (S1 Table), the reasons for which will require clarification in future studies. Correlations of AMP-CMC eQTLs to eQTLs from other GTEx tissues ranged from 0.80 (for brain cortex GTEx brain tissues) to 0.51 (for EBV-transformed lymphocyte cells), with variable agreement on directionality (S2 Table). Focusing analyses on the subset of eQTLs identified by and reported as of identical directionality in other studies further improved specificity of our results (S1 and S2 Tables).

### Mendelian randomization identifies shared genetic effects of brain eQTLs with neurological and psychiatric disease

We conducted MR to determine the shared genetic effects between gene expression (using genetically predicted transcript levels) and 12 neurological and psychiatric diseases (see flow chart in S1 Fig for overview of study design). Our MR analysis included a total of 80,557 Wald ratio tests across 7,137 genes. Of these, 80 Wald ratio effects across 5 out of the 7 psychiatric diseases tested and 4 out of the 5 neurological diseases tested showed evidence of MR at the multiple correction threshold of P<6x10^-7^ (S3 and S4 Tables). The Wald ratio estimates the log odds change in disease risk per standard deviation (SD) change in gene expression relative to the risk allele for the instrumenting SNP. A positive Wald ratio implies a causal relationship between increased expression of the gene and increased disease risk, whereas a negative Wald ratio implies a causal relationship between decreased expression of the gene and increased disease risk. For the psychiatric diseases, we discovered a relationship between 38 genes and schizophrenia, 2 genes and attention deficient hyperactivity disorder, and a single gene each for anorexia, bipolar disorder and major depressive disorder risk. For the neurological diseases we detected a relationship between 16 genes and Parkinson’s disease, 15 genes and Alzheimer’s disease, 4 genes and multiple sclerosis and 2 genes and amyotrophic lateral sclerosis risk. 47 of these 80 signals showed evidence of colocalization between gene expression and disease (PP_4_>70%) indicating that both gene expression and disease trait share the same casual variant in the region (i.e. eQTL instrument is not coincidentally associated with disease SNP due to Linkage Disequilibrium (LD) patterns in the region). We prioritized these co-localizing eQTLs as pointing to the most likely causal genes and substrate for further analyses (Tables 1 and 2). One third (17 genes in total) of the 47 genes co-occurred within the same region of the genome (i.e. instrumented eQTLs used for the different genes were in LD and colocalized with disease, making it difficult to disentangle the causal gene in this region).

**Table 1:** Genes we related with psychiatric traits in the MR and colocalization analysis. Wald ratio (WR) estimate with standard error (SE) and p-value, coloc (posterior probability of colocalization between gene expression and outcome). WR represents the genetically predicted log odds change in risk per standard deviation (SD) change in gene expression.

outcome	exposure gene	SNP	cytogenic	WR	SE	P	coloc
anorexia	*SUOX*	rs1081975	12q13.2	-0.366	0.072	3.00x10^-7^	93.4
bipolar disorder	*RHEBL1*	rs7969091	12q13.12	0.400	0.075	8.63x10^-8^	98.2
major depressive disorder	*PTPN1*	rs718050	20q13.13	-0.195	0.037	1.92x10^-7^	98.4
schizophrenia	*FURIN*	rs4702	15q26.1	-0.237	0.034	2.55x10^-12^	100.0
	*ZNF823*	rs72986630	19p13.2	0.238	0.044	4.90x10^-8^	100.0
	*THOC7*	rs832187	3p14.1	-0.163	0.029	2.57x10^-8^	99.3
	*PTPRU*	rs1498232	1p35.2	-0.256	0.042	1.22x10^-9^	99.0
	*FAM85B*	rs2980436	8p23.1	-0.126	0.023	7.47x10^-8^	98.9
	*KCNQ5*	rs2492966	6q13	0.241	0.045	1.05x10^-7^	98.6
	*LINC00222*	rs9398171	6q21	-0.253	0.046	3.05x10^-8^	98.6
	*CLCN3*	rs10520163	4q33	0.248	0.043	9.19x10^-9^	97.9
	*RERE*	rs2708630	1p36.23	0.193	0.034	1.54x10^-8^	96.7
	*SF3B1*	rs2564383	2q33.1	0.265	0.040	4.82x10^-11^	95.6
	*FTCDNL1*	rs281794	2q33.1	-0.167	0.023	7.75x10^-13^	95.2
	*FAM86B3P*	rs2945253	8p23.1	-0.075	0.015	3.54x10^-7^	94.5
	*ZC3H7B*	rs11090045	22q13.2	0.273	0.051	9.77x10^-8^	93.4
	*INO80E*	rs3814880	16p11.2	0.096	0.016	6.45x10^-10^	92.7
	*GATAD2A*	rs2905435	19p13.11	-0.137	0.025	4.11x10^-8^	92.4
	*NAT8*	rs10179134	2p13.1	0.145	0.028	2.16x10^-7^	91.4
	*AC105749*.*1*	rs11706780	3p22.2	-0.245	0.039	4.17x10^-10^	90.7
	*PCCB*	rs527888	3q22.3	-0.175	0.029	8.79x10^-10^	88.8
	*AC243562*.*2*	rs12905223	15q25.2	-0.113	0.018	4.82x10^-10^	87.5
	*CNTN4*	rs6796313	3p26.3	0.313	0.051	6.76x10^-10^	81.6
	*NMB*	rs56864281	15q25.3	0.307	0.051	1.38x10^-9^	78.8
	*GOLGA2P7*	rs2019611	15q25.2	0.110	0.019	2.64x10^-9^	75.2
	*FES*	rs6224	15q26.1	0.255	0.042	1.58x10^-9^	70.5

**Table 2:** Genes we related with neurological traits detected in the MR and colocalization analysis. WR with SE and p-value, coloc (posterior probability of colocalization between gene expression and outcome). WR represents the genetically predicted log odds change in risk per SD change in gene expression.

outcome	exposure gene	SNP	cytogenic	WR	SE	P	coloc
Alzheimer’s disease	*CR1*	rs679515	1q32.2	0.274	0.028	2.15x10^-23^	99.6
	*CCDC6*	rs1171830	10q21.2	0.222	0.039	1.22x10^-8^	99.5
	*ACE*	rs4295	17q23.3	-0.153	0.028	3.37x10^-8^	99.0
	*TSPAN14*	rs7097656	10q23.1	0.131	0.025	2.64x10^-7^	98.5
	*KAT8*	rs12597511	16p11.2	-0.133	0.023	6.18x10^-9^	95.5
	*ZNF646*	rs8050894	16p11.2	-0.216	0.037	3.53x10^-9^	93.7
	*CCNT2-AS1*	rs766271	2q21.3	-0.151	0.030	5.06x10^-7^	91.7
	*PRSS36*	rs55667375	16p11.2	-0.135	0.022	1.66x10^-9^	83.6
	*AC012146*.*1*	rs56377155	17p13.2	-0.144	0.023	5.62x10^-10^	77.0
amyotrophic lateral sclerosis	*SCFD1*	rs229184	14q12	0.141	0.028	5.14x10^-7^	92.6
	*G2E3*	rs229243	14q12	-0.201	0.040	5.94x10^-7^	92.2
multiple sclerosis	*MPV17L2*	rs62120364	19p13.11	-0.477	0.087	4.34x10^-8^	99.9
	*TTC34*	rs4310388	1p36.32	-0.487	0.066	2.26x10^-13^	99.8
	*IQCB1*	rs6438665	3q13.33	0.118	0.022	8.08x10^-8^	90.9
	*SLC30A7*	rs6681932	1p21.2	-0.357	0.067	9.85x10^-8^	82.9
Parkinson’s disease	*GRN*	rs5848	17q21.31	-0.242	0.047	2.18x10^-7^	98.4
	*STX4*	rs7184567	16p11.2	0.318	0.051	6.13x10^-10^	95.9
	*GPNMB*	rs858239	7p15.3	0.121	0.017	2.37x10^-13^	95.5
	*HSD3B7*	rs7196726	16p11.2	-0.301	0.051	2.71x10^-9^	89.8
	*KLHL7-DT*	rs73272053	7p15.3	-0.295	0.042	2.69x10^-12^	77.2
	*AC135050*.*3*	rs35713203	16p11.2	-0.235	0.046	3.74x10^-7^	72.2

#### Shared genetic effects between brain gene expression and psychiatric diseases

**Fig 1:**
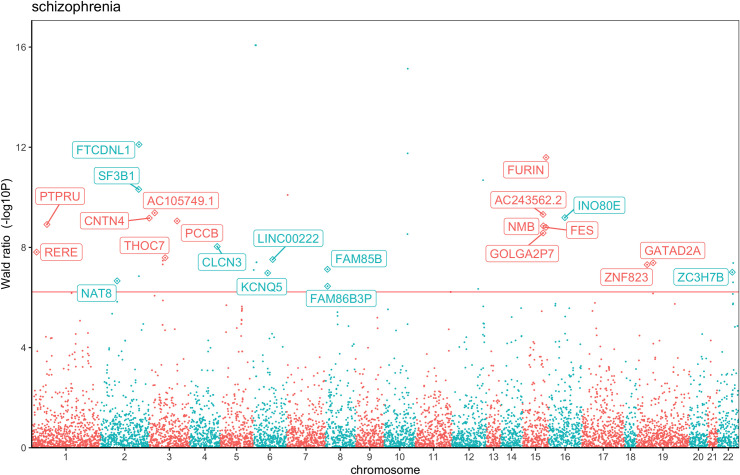
Manhattan plot showing the MR relationships between gene expression changes and schizophrenia risk. Chromosomal position is on the x-axis and p-value for the Wald ratio estimate (-log10 scaled) is on the y-axis. The prioritised genes which passed the multiple correction threshold (solid red line) and colocalization analysis (PP_4_>70%) are annotated as diamonds and labelled with the gene name. *FAM86B3P* and *FAM85B* genes on chromosome 8 and *AC243562*.*2*, *GOLGA2P7* and *NMB* genes and *FURIN* and *FES* genes on chromosome 15 are located close together (in LD with each other), challenging attribution of the causal gene at these loci.

MR and colocalization analyses identified 23 potentially causal genes with evidence of a shared genetic effect between gene expression (eQTL) and schizophrenia risk (Table 1). Of these, 12 genes showed a positive direction of Wald ratio effect, indicating a relationship between increased gene expression and increased schizophrenia risk (*SF3B1*, *IN080E*, *CNTN4*, *NMB*, *FES*, *GOLGA2P7*, *CLCN3*, *RERE*, *ZNF823*, *ZC3H7B*, *KCNQ5*, and *NAT8*). 11 genes showed a negative direction of Wald ratio effect indicating a relationship between decreased gene expression and increased schizophrenia risk (*FTCDNL1*, *FURIN*, *AC243562*.*2*, *AC105749*.*1*, *PCCB*, *PTPRU*, *THOC7*, *LINC02210*, *GATAD2A*, *FAM85B* and *FAM86B3P*). Notably, 7 out of the 23 genes reside in close genomic proximity, challenging attribution of causality for schizophrenia risk (Fig 1). Instruments for *AC243562*.*2* and *GOLGA2P7* are in high LD with each other (r^2^ = 0.91) and in moderate LD with eQTLs impacting *NMB* (r2 ≈ 0.55 with each instrument). Instruments for the nearby *FURIN* and *FES* genes are in moderate LD with each other (r^2^ = 0.67). Likewise, eQTLs for *FAM86B3P* and *FAM85B* are in high LD (r^2^ = 0.83), limiting resolution of MR analyses. For the remaining 6 psychiatric diseases, a positive Wald ratio effect was observed for *RHEBL* expression and bipolar disorder, and negative Wald ratio effects for *SUOX* expression and anorexia risk, and for *PTPN1* expression and major depressive disorder risk.

#### Shared genetic effects between gene expression and neurological diseases

**Fig 2:**
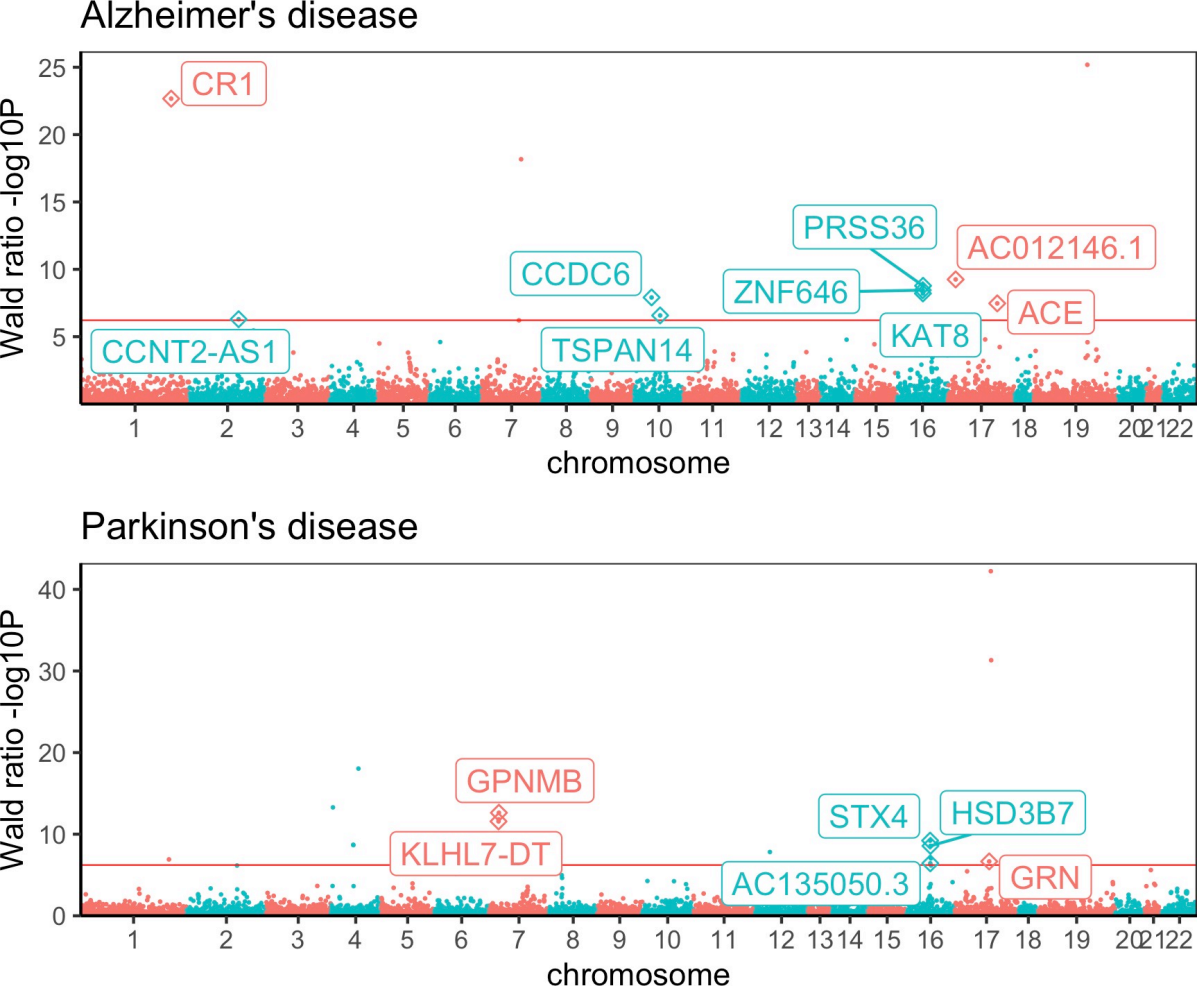
Manhattan plots showing the MR relationships between gene expression changes and Alzheimer’s disease and Parkinson’s disease outcomes. Chromosomal position is on the x-axis and p-value for the Wald ratio estimate (-log10 scaled) is on the y-axis. The prioritised genes which passed the multiple correction threshold (solid red line) and colocalization analysis (PP_4_>70%) are annotated as diamonds and labelled with the gene name. For Alzheimer’s disease the *ZNF646*, *KAT8* and *PRSS36* genes on chromosome 16 are in LD, and for Parkinson’s disease the *GPNMB* and *KLHL7-DT* genes on chromosome 7 and the *STX4*, *AC135050*.*3* and *HSD3B7* on chromosome 16 are in LD, challenging attribution of the causal gene at these loci.

MR and colocalization analyses further identified 21 potentially causal genes with evidence of a shared genetic effect between gene expression and five neurological diseases tested (Table 2). We detected a positive Wald ratio effect between the expression of three genes (*CR1*, *CCDC6*, *TSPAN14*), and a negative Wald ratio effect between the expression of 6 genes (*AC012146*.*1*, *PRSS36*, *ZNF646*, *KAT8*, *ACE*, *CCNT2-AS1*) and the risk for Alzheimer’s disease. Of these, instruments for *ZNF646* and *KAT8* are in high LD (r^2^ = 0.85), and for *PRSS36* in relatively low LD (r^2^≈0.2 with each instrument) (Fig 2). We further found a positive Wald ratio effect between the expression of two genes (*GPNMB*, *STX4*) and Parkinson’s disease risk, and a negative Wald ratio effect between the expression of four genes (*KLHL7-DT*, *HSD3B7*, *GRN*, *AC135050*.*3)* and Parkinson’s disease risk. Instruments for *STX4*, *AC135050*.*3* and *HSD3B7* are in high LD (r^2^>0.85), and for *KLHL7-DT* and *GPNMB* are in moderate LD (r^2^ = 0.64) (Fig 2). Moreover, we identified Wald ratio effects between four genes (*IQCB*, *TTC34*, *MPV17L2* and *SLC30A7)* and multiple sclerosis risk, and effects between two genes (*SCFD1*, *G2E3*) and amyotrophic lateral sclerosis risk. *SCDF1* and *G2E3* instruments are in high LD (r^2^ = 0.99).

#### Identification of pleiotropic effects across neurological and psychiatric diseases

To assess whether the neurological and psychiatric diseases analysed in this study share pleiotropic relationships, we next queried for evidence of common Wald ratio effects at a less stringent significance threshold of P<0.05 for our 47 most likely causal candidate genes. 141 of the 551 Wald ratio estimates showed a relationship with a second disease at this cut-off. Of these, nine genes also passed colocalization (PP_4_>70%) with this alternative disease. This included six of the schizophrenia genes (*GOLGA2P7*, *NMB*, *AC105749*.*1*, *FES*, *FURIN*, *FTCDNL1*), both of the amyotrophic lateral sclerosis genes (*SCFD1* and *G2E3)*, and one of the genes related with Parkinson’s disease risk (*GRN*) (Table 3). For instance, expression levels of *G2E3* and *SCFD1* not only appear to causally effect the risk for amyotrophic lateral sclerosis but also attention deficient disorder, albeit with alternative directionalities. Wald ratio effects with identical directionality for two diseases were observed for *GRN* with Parkinson’s disease and Alzheimer’s disease; for *AC105749*.*1*, *GOLGA2P7* and *NMB* with schizophrenia and bipolar disorder; and *FES* and *FURIN* with schizophrenia and major depressive disorder. Permutation testing suggested that the likelihood to observe these shared genetic effects by chance was very low (empirical p-value<1x10^-4^; Methods).

**Table 3:** Genes which shared MR and colocalization evidence across the psychiatric and neurological outcomes we tested. The outcome detected in our main MR is highlighted in bold. Amyotrophic lateral sclerosis (ALS), attention deficient disorder (ADHD), Parkinson’s disease (PD), Alzheimer’s disease (AD), schizophrenia (Sz), multiple sclerosis (MS), bipolar disorder (BD), and major depressive disorder (MDD).

gene	SNP	cytogenic	outcome	WR	SE	P	coloc
*G2E3*	rs229243	14q12	**ALS**	-0.201	0.040	5.94x10^-7^	92.2
*G2E3*	rs229243	14q12	ADHD	0.136	0.040	6.47x10^-4^	71.4
*SCFD1*	rs229184	14q12	**ALS**	0.141	0.028	5.14x10^-7^	92.6
*SCFD1*	rs229184	14q12	ADHD	-0.092	0.027	8.16x10^-4^	70.6
*GRN*	rs5848	17q21.31	**PD**	-0.242	0.047	2.18x10^-7^	98.4
*GRN*	rs5848	17q12.31	AD	-0.145	0.034	1.60x10^-5^	97.9
*AC105749*.*1*	rs11706780	3p22.2	**Sz**	-0.245	0.039	4.17x10^-10^	90.7
*AC105749*.*1*	rs11706780	3p22.2	BD	-0.370	0.085	1.33x10^-5^	97.9
*FTCDNL1*	rs281794	2q33.1	**Sz**	-0.167	0.023	7.75x10^-13^	95.2
*FTCDNL1*	rs281794	2q33.1	MS	0.158	0.047	7.10x10^-4^	98.3
*GOLGA2P7*	rs2019611	15q25.2	**Sz**	0.110	0.019	2.64x10^-9^	75.2
*GOLGA2P7*	rs2019611	15q25.2	BD	0.130	0.038	6.87x10^-4^	73.0
*NMB*	rs56864281	15q25.3	**Sz**	0.307	0.051	1.38x10^-9^	78.8
*NMB*	rs56864281	15q25.3	BD	0.332	0.112	2.97x10^-3^	86.9
*FES*	rs6224	15q25.2	**Sz**	0.255	0.042	1.58x10^-9^	70.5
*FES*	rs6224	15q25.2	MDD	0.120	0.031	1.42x10^-4^	76.8
*FURIN*	rs4702	15q26.1	**Sz**	-0.237	0.034	2.55x10^-12^	100.0
*FURIN*	rs4702	15q26.1	MDD	-0.096	0.025	1.34x10^-4^	78.4

### Follow up MR analysis on selected instruments to assess pleiotropy, tissue specificity and reverse causation

For the 47 signals identified from our primary MR analysis we conducted a series of follow-up analyses to assess the validity of our instruments.

#### Assessment of pleiotropy phenome-wide: enrichment for association with brain-related traits with no evidence of horizontal pleiotropy

It is possible that undetected horizontal pleiotropy may explain the MR relationship between gene expression and disease observed in our study. This occurs when the instrument is associated with risk factors that exert their effect on disease through distinct pathways, which is in contrast to vertical pleiotropy, where the instrument is associated with risk factors that act through the same pathway, and does not violate the assumptions of MR [17,18]. We therefore conducted a phenome-wide assessment on the 47 selected instruments in order to assess the extent of pleiotropy and risk of violating MR assumptions. For this, a total of 9,441 Wald ratio tests were computed across 219 disease and risk factor traits (see Methods). 133 Wald ratios showed evidence of a relationship at the multiple testing correction threshold (P<6x10^-6^) and an additional 1,037 Wald ratios showed weaker evidence of a relationship (P<0.05). Of these MR effects, 103 showed evidence of colocalization between gene expression and the selected trait (PP_4_>70%), which consisted of 64 from the 133 Wald ratios passing the threshold of P<6x10^-6^ and an additional 39 from the 1,037 Wald ratios passing the threshold of P< 0.05. 1,549 (16%) of the Wald ratios pertained to brain-related traits (defined as neurological, psychiatric, education, personality and behaviour categories), of which 41 colocalised: 29 Wald ratios at P<6x10^-6^ (29/1549 = 1.87%) and an additional 12 Wald ratios at P <0.05 (41/1549 = 2.65%). 7,892 (84%) of the Wald ratios corresponded to other non-brain related traits of which 62 colocalised: 35 Wald ratios at P<6x10^-6^ (35/7892 = 0.44%) and an additional 27 Wald ratios at the P<0.05 threshold (62/7892 = 0.79%). Overall, the phenome-wide MR analysis showed evidence for enrichment of colocalization between gene expression and brain-related traits relative to colocalization with other traits (Fisher’s exact test p-values = p<0.0001 for both the P<6x10^-6^ and P<0.05 thresholds), which suggests that much of the pleiotropy present is likely to be vertical (i.e. potentially acting through the same brain related pathway). Colocalization was detected with an additional non-brain trait for 20 of the 47 genes at the P<6x10^-6^ and another 4 genes at the P<0.05 threshold (S5 Table). Although this shows that non-brain specific effects are present none of the traits appear to open horizonal pleiotropic pathways which would violate the MR. We found evidence of non-brain specific effects (Wald Ratio P<0.05 and coloc PP_4_>70%) for two of the genes (*FES* and *FURIN)* we had previously identified as having pleiotropy effects across the primary outcomes in our main MR. Increased *FES* expression colocalised with increased birth weight, increased ovarian cancer risk and decreased blood pressure along with decreased risk of hypertension and other related cardiovascular events and increased *FURIN* colocalised with decreased birth weight and increased angina risk (S5 Table). Our phenome-wide analysis suggests there is a limited scope for horizontal pleiotropy to affect our MR results. However, it is challenging to distinguish horizontal from vertical pleiotropy without an intricate and complete understanding of the pathways, therefore latent pleiotropic factors could still be confounding certain MR estimates.

#### Molecular pleiotropy is pervasive

Another potential source of horizontal pleiotropy could be through instrument association with the expression of multiple cis or trans genes (molecular pleiotropy). We observed in our main MR analysis that several of the genes resided within the same genomic region, indicating potential for molecular pleiotropy. However, our MR analysis will miss genes where the eQTL could not be included as an instrument due to the SNP not being robustly associated enough (P<5x10^-8^) or not finding the SNP in the outcome. Therefore, in order to more extensively evaluate pleiotropy within the cis-region, the 47 instruments used for the genes identified in our main MR were looked up across the gene transcripts available in AMP-CMC (without employing a p-value threshold). A total of 1,482 SNP-gene expression effect estimates were extracted. Of these 143 SNPs were associated with gene expression effects at a multiple testing correction threshold of P<3.4x10^-5^, from which 87 also passed colocalization (PP_4_>70%) with the primary outcome. 23 of the 47 instruments showed evidence of colocalization between the primary outcome and more than one gene (S6 Table). In terms of the degree of molecular pleiotropy this consisted of 11 instruments associated with 3 genes, 10 instruments with 2 genes, and 2 instruments with 5 different genes. For the other 24 instruments which colocalised with the expression of a single gene only, there is less ambiguity regarding the causal variant and therefore potential genetic pathways involved which strengthen these as drug target candidates.

#### Evidence of tissue specific effects

**Fig 3:**
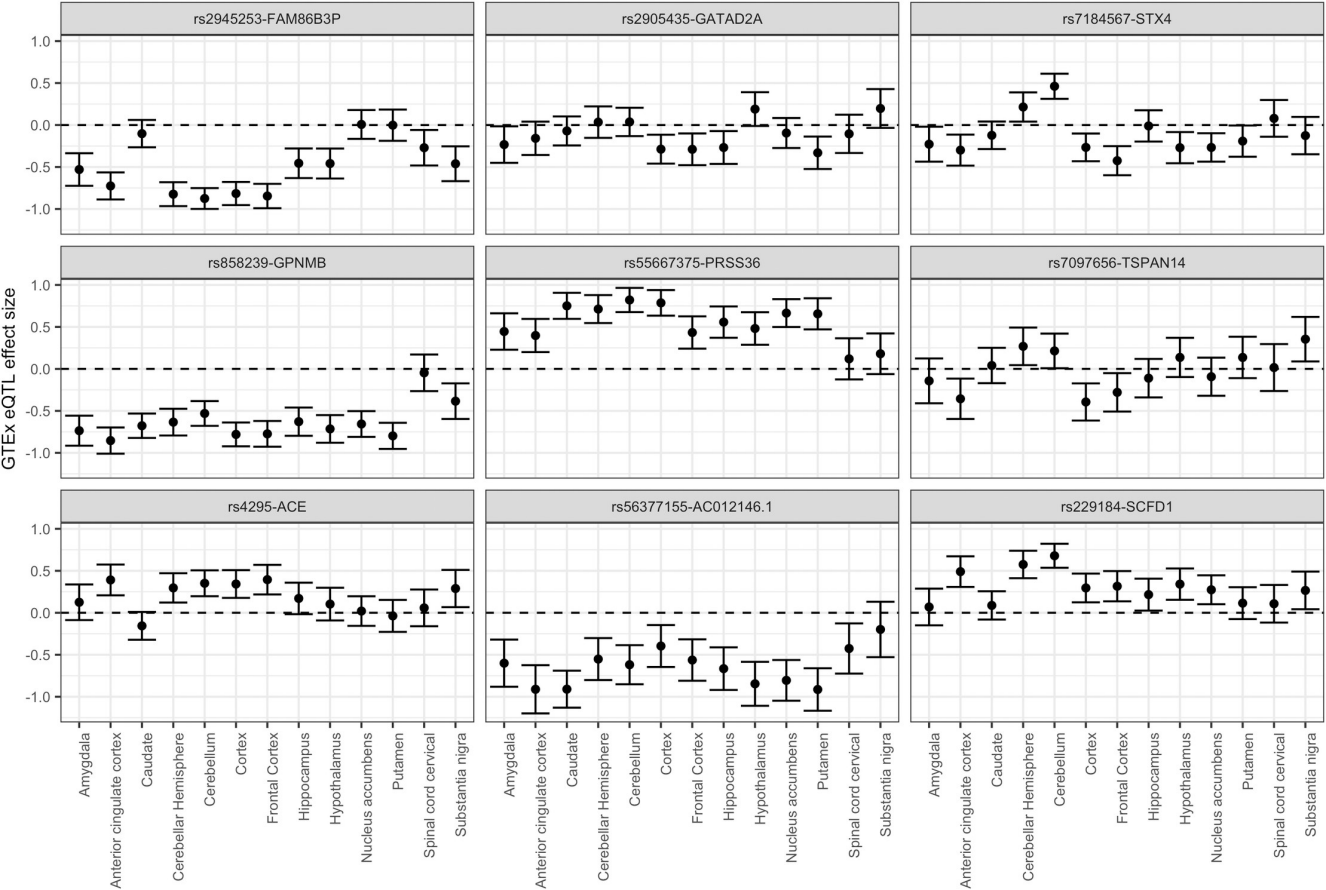
Error bar plot (95% confidence interval around the eQTL effect is displayed) comparing gene expression effects across the 13 brain tissues available in GTEx version 7. 9 of our 47 prioritised genes showed statistical evidence of effect size heterogeneity across the brain tissues. *STX4* and *TSPAN14* genes showed evidence of differing direction of gene expression effect for certain brain tissues.

We investigated whether our top MR findings would be generalisable to other brain tissues by looking up the instruments within the 13 brain tissues available in GTEx version 7. 44 of our 47 instruments were also present in GTEx, all SNPs except one (rs9398171-*LINC00222* instrument) had effect estimates available across every brain tissue. 167 tissue effects from 566 effects available in total, across 27 of the 44 instruments, showed evidence of eQTL association at a multiple-testing corrected threshold (P < 9x10^-5^, *α* = 0.05) in the GTEx tissues (S7 Table). All these associations except one (rs7184567-*STX4* association in Cerebellum tissue) had the same direction of effect as our instrument. As we would expect, the overall correlation between the eQTL effects in our study and GTEx (considering all 13 brain tissues grouped together) was moderately high (r = 0.74, 95% CI = [0.71, 0.78]). Correlations were also computed within the separate tissues and were of a similar magnitude. 9 of the instruments showed evidence of eQTL effect size heterogeneity (Cochran’s Q test; P < 0.001) across GTEx tissues, which consisted of rs2945253-*FAM863BP* and rs2905435-*GATA2DA* associations with schizophrenia, rs7184567-*STX4* and rs858239-*GPNMB* associations with Parkinson’s disease; rs55667375-*PRSS36*, rs7097656-*TSPAN14*, rs4295-*ACE* and rs56377155-*AC012146*.*1* associations with Alzheimer’s disease; and the rs229184-*SCFD1* association with amyotrophic lateral sclerosis (S8 Table). On inspection of the error bar plots (Fig 3), we found two of the instruments (rs7184567-*STX4* and rs7097656-*TSPAN14*) displayed a difference in direction of effect between certain brain tissues. rs7184567-*STX4* eQTL showed evidence (P<0.05) of increased expression in the cerebellar hemisphere and cerebellum tissues and decreased expression in the amygdala, anterior cingulate cortex, cortex, frontal cortex, hypothalamus, nucleus accumbens, and putamen tissues. rs7097656-*TSPAN14* eQTL showed evidence (P<0.05) of increased expression in the cerebellar hemisphere, cerebellum, and substantia nigra tissues, and decreased expression in the anterior cingulate cortex, cortex and frontal cortex tissues. As an additional analysis, we then looked up our brain instruments in eQTLGen to determine if blood tissue would have been a reasonable proxy for the effects we observed. 42 of our brain instruments were found in the eQTLGen study. Correlation of the estimates between our brain study and eQTLGen was moderate (r = 0.56, 95% CI = [0.30,0.73)]. 18 of the 42 instruments were genome-wide significant (P<5x10^-8^) in eQTLGen and showed evidence of colocalization (PP_4_>70%) with the same gene’s expression measured in our brain study (S9 Table). All 18 of the instruments apart from two, the rs229184-*SCFD1* and rs7184567-*STX4* associations, had the same direction of expression effect as our brain instrument. We also re-performed MR analysis instrumenting on eQTLs available within eQTLGen in order to establish whether additional genes could be reliably discovered using blood tissue as a proxy. A total of 41,157 independent SNPs across 16,190 genes were available as instruments at the P<5x10^-8^ cut-off in eQTLGen. eQTLGen allowed for the instrumentation of many other genes with 10,754 of the 16,190 genes (66.4%) not intersecting with the genes we were able to instrument in the AMP-CMC MR analysis (S10 Table). 449 instruments (n = 417 genes) at the P< 6x10^-7^ and 30,245 instruments (n = 11,818 genes) at the P<0.05 cut-off showed evidence of a MR effect from the 373,176 MR tests conducted in total across the 12 primary outcomes tested (full eQTLGen MR results available at GitHub repository denisbrd/braineQTLMR_datasets). We then matched the MR effect estimates for these eQTLGen instruments with the AMP-CMC MR estimates (n = 7,689 instruments across 7,136 genes) and found that a large proportion of the effects had discrepant directions: at the P< 6x10^-7^ cut-off, 50 from the 110 MR effects shared between the studies (45.5%) were in the opposite direction, and at the P<0.05 cut-off 4,068 from 10,494 shared MR effects (38.9%) were in the opposite direction.

#### No evidence of reverse causation

As the genetic instruments were selected from a meta-analysis which contained post-mortem samples collected from the brain tissue of Alzheimer’s disease patients, it is possible that some of the gene expression changes are a consequence of Alzheimer’s disease pathogenesis rather than being cause of a neurological disease. We therefore examined whether our MR effects were in the correct direction using reverse MR and Steiger filtering. In reverse MR, we assessed the effect of the outcome on gene expression, using SNPs associated with the primary outcome at P<5x10^-8^ as instruments. A total of 13,282 SNPs was retrieved from the primary outcome GWASs, which reduced to 180 independent SNP instruments after LD clumping. These consisted of 83 SNPs associated with schizophrenia, 28 with Alzheimer’s disease, 27 with Parkinson’s disease, 26 with multiple sclerosis, 6 with amyotrophic lateral sclerosis, 5 with major depressive disorder, 4 with bipolar disorder and one with anorexia nervosa. After harmonisation with SNP effects retrieved from eQTLGen, it was possible to conduct reverse MR for 41 of the 47 genes identified in the primary MR. Little evidence of MR relationships in the reverse direction (from outcome to gene expression) was detected for any of the genes (S11 Table). Steiger filtering was performed on the brain eQTL dataset from our study, and little evidence of reverse causation was detected from this analysis as well (S12 Table).

### MR outcomes inform drug discovery

We next assessed whether the genes prioritized by MR might serve as promising targets for drug discovery. For this, we assessed each of the 47 genes causally linked to neurologic or psychiatric disease using the following three criteria: (i) evidence for an “allelic series” with coding variants reported to cause Mendelian diseases with neurological or behavioural phenotypes; (ii) evidence for being the target of a marketed or clinically tested drug; and (iii) a satisfying safety profile in our phenome-wide MR analyses. Of the 47 genes, five (*RERE*, *KCNQ5*, *SUOX*, *ACE* and *GPNMB)* fulfilled our criteria as strong candidates for drug development (Tables S13 and 4). Our MR results suggest that inhibition of *RERE* and *KCNQ5* could be used to treat schizophrenia and *GPNMB* to treat Parkinson’s disease whereas promotion of *SUOX* could be used to treat Anorexia and *ACE* to treat Alzheimer’s disease. For instance, mutations in three genes our MR analyses linked to schizophrenia (*RERE*, *KCNQ5*) or anorexia (*SUOX*), respectively, are reported to cause monogenic diseases. Loss-of *RERE* function results in an early-onset neurodevelopmental disorder with anomalies in the brain, eye and heart (OMIM 605226) [19,20], and haploinsufficiency for *RERE* variants with assumed milder impact are believed to contribute to neurological abnormalities in adults [21]. Mutations in *KCNQ5* cause an autosomal-dominant mental retardation syndrome (OMIM 607357). *KCNQ5* is part of the transmembrane voltage-gated potassium channel gene family involved in the regulation of the conduction of electrical signals across nerve and muscle cells [22,23]. Lehman et al. identified both gain (increased nerve cell excitability) and loss of function (decreased nerve cell excitability) autosomal dominant mutations in the *KCNQ5* gene after conducting exome sequencing on families with intellectual disability and epilepsy [24]. *SUOX* encodes the sulphite oxidase enzyme responsible for breaking down sulfuric amino acids accumulation of which is neurotoxic. Autosomal-recessive deficiency for this enzyme results in Isolated Sulfite Oxidase Deficiency (ISOD) which presents as a neurodevelopmental disorder with motor and behavioural signs (OMIM 272300).

Three of the 47 genes had been considered earlier for drug development and are listed as targets for approved medicines or drugs tested in clinical trials. For instance, Dalfampridine is a *KCNQ5* inhibitor that has been approved to improve mobility in multiple sclerosis patients, and *KCNQ5* inhibitors [25] have been explored also for other neurological conditions including amyotrophic lateral sclerosis, Gullian-Barre Syndrome, unipolar depression and cerebral palsy (Open Target annotations). Similarly, *GPNMB* inhibitors have been explored for various types of cancer, for instance the monoclonoal antibody glembatumab (CDX-011) in Phase 2 trials for melanoma and breast cancer [26,27]. And ACE inhibitors are a well-established approved class of drugs for the treatment of hypertension, heart failure and diabetes mellitus.

We conducted an MR-PheWAS analysis to assess whether targeting the five genes that showed either an “allelic series” or were previously reported as drug targets would be safe (Table 4). Substantial new toxicity signals were identified for none of the five genes. However, consistent with the known pharmacological effect of ACE inhibitors to lower blood pressure, increased *ACE* expression colocalised with increased hypertension risk which might limit exploration of this target to treat or prevent Alzheimer’s disease. Also, an increase in *SUOX* expression as it might be desirable to treat anorexia, colocalised with reduced hypothyroidism risk and additionally an elevated risk for rheumatoid arthritis and hypertension, warranting further safety investigations in follow up studies. Interestingly, expression of *RERE* also colocalized with heel bone mineral density, suggesting that in addition to schizophrenia *RERE* inhibitors (decreasing expression of *RERE*) might potentially be beneficial in osteoporosis (increasing heel bone BMD) and eventually other non-CNS traits, which will need to be clarified in future studies.

**Table 4:** Genes we prioritised as drug targets that had MR and colocalization evidence with other indications. Gene expression-disease relationship identified in the main MR (instrument-gene-indication): Alzheimer’s disease (AD), Schizophrenia (Sz), anorexia (AN), suggested direction of drug action according to MR effect (inhibition or promotion of gene expression), other indications detected with the same gene in our MR-PheWAS (other indication), Wald ratio (WR), SE and P-value of MR effect and colocalization probability PP_4_ (coloc).

instrument-gene-indication	drug mechanism	other indication	WR	SE	P	coloc
rs4295-*ACE*-AD	promotion (negative MR effect)	Non-cancer illness code self-reported: hypertension	0.024	0.003	3.57x10^-15^	99.0
rs2708630*-RERE-*Sz	inhibition (positive MR effect)	Heel bone mineral density (BMD) T-score automated	-0.080	0.010	2.17x10^-16^	97.1
		Diastolic blood pressure automated reading	0.054	0.008	2.15x10^-12^	96.6
		Systolic blood pressure automated reading	0.050	0.008	1.58x10^-10^	96.7
		Age at menarche	0.081	0.021	1.50x10^-4^	97.8
rs1081975*-SUOX-*AN	promotion (negative MR effect)	Non-cancer illness code self-reported: hypothyroidism/myxoedema	-0.008	0.001	1.20x10^-10^	87.0
		Rheumatoid arthritis	0.199	0.033	2.23x10^-9^	97.9
		Non-cancer illness code self-reported: hypertension	0.011	0.003	3.06x10^-5^	74.9

## Discussion

In this study, we used a combination of two sample MR and colocalization analysis to infer potential causal relationships between expression of genes measured in brain tissues and 12 neurological and psychiatric conditions. MR was used to mitigate the confounding that may arise when assessing associations between gene expression and disease, and colocalization was used to verify that the eQTL instrument used in the MR was not coincidentally associated with both traits (i.e. eliminate the possibility of the MR effect arising due to alternative causal variants in LD). This two-step approach to causal gene identification is important since expression of many genes with MR effects does not colocalize with association signals for the outcome trait. For instance, a previous two sample MR study [9] showed that many of the genes detected in their MR did not subsequently pass the HEIDI test (around 65%) used to distinguish between genuine pleiotropy and association through LD. In our current study, 47 eQTLs showed evidence of a shared genetic effect with neurological or psychiatric disease outcomes through MR and colocalization, suggesting a functional role for the genes in disease pathogenesis. 16 of these eQTLs had only a suggestive association (5x10^-8^<P<5x10^-5^) in the respective outcome GWAS, which suggests that integrating eQTL evidence may help substantiate GWAS findings.

For eQTLs prioritized by MR, we investigated whether there was evidence for shared effects across the different neurological or psychiatric outcomes analysed, opening the possibility for identifying intervention points for novel drugs with cross-indication potential. No evidence for sharing of genetic effects at our multiple testing corrected P value threshold was detected. However, as we expected the genes we identified in our main MR to share outcome effects more often than a random gene we also looked for evidence of shared relationships at a less stringent threshold of P<0.05, and found some weaker evidence of shared MR effects between schizophrenia, amyotrophic lateral sclerosis, and Parkinson’s disease genes. *GRN* emerged as a particular promising target from our analyses since it was prioritised in our main MR analysis for Parkinson’s disease, but also showed a suggestive relationship with Alzheimer’s disease risk. *GRN* encodes the progranulin protein, that is highly active in brain cells and plays an important role in the survival of neurons. Autosomal dominant mutations in this gene have been found to result in frontotemporal dementia via haploinsufficiency, whereby the shortage of progranulin causes a build-up of TDP-43 aggregates in the brain resulting in premature cell death [28]. Mutations in this gene have also been identified in patients diagnosed with Alzheimer’s disease [29]. A whole genome-wide sequencing study [30] discovered evidence of enrichment of rare deleterious variants in the *GRN* gene amongst Parkinson’s disease cases relative to European controls.

We performed a systematic comparison of our instrument effects against other eQTL datasets in order to evaluate the generalisability of our MR findings. We found that a significant proportion of our instruments showed the inverse direction of effect when compared against blood tissue effects in eQTLGen and across a range of other non-brain related tissue effects in GTEx. We then performed the comparison on effect sizes for the instruments belonging to our top 47 gene-expression disease MR relationships. Heterogeneity tests revealed evidence of differing eQTL effect sizes for *FAM86B3P*, *STX4*, *PRSS36*, *GPNMB*, *TSPAN14*, *SCFD1*, *ACE*, *GATAD2A* and *AC012146*.*1* genes across the 13 brain tissues in GTEx. In particular, *STX4* and *TSPAN14* showed opposing directions of expression effects for certain tissues, which could result in drawing differing MR conclusions regarding direction of effect. Under half of the brain instruments for our top findings were also suitable instruments in eQTLGen, which indicates that disease relevant loci would have been missed if the study had relied on using a blood tissue as a proxy. In the MR analysis we conducted on eQTLGen instruments, we were able to detect many additional genes related to our diseases not picked up in our brain eQTL analysis, however the shared MR effects with our brain study tended to have a different causal direction. This argues that future MR studies should be conducted in disease relevant tissues to ensure the correct direction of causality. When there is uncertainty regarding the correct tissue, then it is important to examine instrument effects across a range of applicable tissues rather than relying on a single tissue. Studies which seek to enhance statistical power through combining eQTL effects across tissues, such as the MeCs meta-analysis method across brain tissues [10], will require the assumption of homogeneity of effect across tissues for all loci to be checked beforehand.

We examined the potential for our top MR findings to inform drug discovery and identified three genes (*RERE*, *KCNQ5*, *SUOX*) in which high-impact mutations are reported to cause Mendelian CNS diseases, suggestive of function-phenotype effects observed for “allelic series” genes [1]. Moreover, three genes (*ACE*, *GPMNB*, *KCNQ5)* have already being explored as drug targets in clinical trials or are the targets of marketed drugs, proposing existing therapies that could be explored for CNS conditions.

ACE inhibitors were found to have strong evidence of a causal relationship with increased Alzheimer’s disease risk in a previous MR study conducted to investigate the use of antihypertensive drugs to treat the disease [31]. However, mice models have demonstrated that increased expression of *ACE* helps to prevent cognitive decline and to protect against amyloid Beta-protein deposits [32], which if they build up in a brain are a risk factor for the development of Alzheimer’s disease. Therefore, we recommend that although we found that promotion of ACE could help to treat Alzheimer’s disease, due to the risks associated with the consequent increased blood pressure this action would require further functional studies to evaluate safety.

Our MR suggested that *GPNMB* inhibitors could be re-purposed to treat Parkinson’s disease. The role of GPNMB is already well-established in tumour progression, where over-expression of the gene suppresses the immune response to tumour growth [33]. Evidence also exists that supports a similar pathogenic role for this gene in Parkinson’s disease. Both *GPNMB* gene expression and protein levels have been shown to be elevated within brain regions of Parkinson’s disease patients, and functional follow-up in these same studies indicated that *GPNMB* may act through suppression of the inflammatory response [34,35]. Our *GPNMB* MR finding was consistent with a transcriptome-wide association study (TWAS) conducted by Li et al [36] on the Nalls et al [37] Parkinson’s disease GWAS, who also found that increased *GPNMB* expression in dorsolateral prefrontal cortex and peripheral monocytes was associated with increased disease risk.

MR further suggests that *KCNQ5* inhibitors could be re-purposed to treat schizophrenia. Potassium channel blockers are known to be effective in the treatment of multiple sclerosis [25], and several studies suggest that genes within this family would make strong candidates for the treatment of symptoms associated with schizophrenia [38]. For example, a SNP within the *KCNH7* gene was demonstrated to be involved in modulating the treatment response of schizophrenics to the antipsychotic drug risperidone, therefore the gene was recommended as a novel drug target by this study [39].

We decided to be relatively conservative with our cut-off used to select our instruments (P<5x10^-8^) and selection of our top MR findings to avoid false positive MR findings. Although, two sample MR studies will frequently select SNPs at the P<5x10^-8^ as instruments, less stringent thresholds have been used for eQTL studies. For example, to investigate brain tissue-specific gene expression effects on schizophrenia, a previous MR study [9] conducted on a brain tissue eQTL dataset (n = 134) [40] used a less stringent P value threshold of P<1.6x10^-3^. Therefore, it is possible that we have missed reporting some genes that have a true casual effect. In contrast, for our colocalization analysis, we decided to use a relatively liberal probability cut-off of PP_4_ > 70% to avoid wrongly eliminating potentially interesting and important loci (i.e. reduce false negative rate). For example, in the original method publication [41] considered a cut-off of PP_4_ > 75% to be strong evidence of colocalization and other studies have used PP_4_>80% as the cut-off to determine colocalization between traits[12,42]. 6 from 47 of our prioritised genes colocalized with a PP_4_ of between 70% to 80%: *NMB*, *GOLGA2P7*, *FES* genes with schizophrenia, *KLHL7-DT* and *AC135050*.*3* genes with Parkinson’s Disease, and the *AC012146*.*1* gene with Alzheimer’s disease. These findings should therefore be considered as having more borderline evidence of colocalization. It is also possible that regions may fail to colocalize due to lack of statistical power and multiple causal variants in the region, which may obscure association peaks that would colocalize after conditional analysis is conducted across the region.

A limitation of our MR study, which is true of MR studies on QTLs in general, is the sparsity of SNP instruments available within most of the genomic regions. For the MR analysis, only a small fraction of genes could be instrumented by more than one SNP (of which the vast majority could only be instrumented by two SNPs), therefore the type of MR and sensitivity analysis that could be conducted was limited. Due to the lack of single SNP instruments within genes it was not possible to achieve substantially stronger instrumentation via the Inverse Variance Weighted (IVW) method (where Wald ratio effects are pooled across the same gene transcript as a weighted average to obtain an MR effect, full IVW results available at GitHub repository denisbrd/braineQTLMR_datasets) and to conduct many of the sensitivity tests to assess the impact of heterogeneity, invalid instruments and pleiotropy on the MR inference [43]. For example, for around half of our top findings’ instruments, we detected shared association with other eQTLs for genes in the cis-region, making it difficult to prioritise the causal gene at this locus (i.e. imperfect drug target specificity) and also raising the possibility that these genes could be confounded via horizontal pleiotropy if genes acted through different molecular pathways. Reassuringly, we identified no such issues with the genes which we prioritised as drug targets as there was no evidence of shared association with the expression of other genes in their genomic region.

In conclusion, our study demonstrates the utility of using transcriptome-wide MR and colocalization analysis in brain tissue to identify drug targets for psychiatric and neurological diseases. However, it is important to note that our MR inference was based on outcomes which captured the incident risk of the disease, which relates to factors associated with onset of disease. As such targeting our findings with drugs may be useful in prevention of disease but it is not clear whether this may be effective in preventing progression of the disease as well. GWASs are increasingly becoming available on disease progression traits which can be used in future MR studies to confirm whether susceptibility loci are also involved in disease progression and can be targeted by drugs to slow this process. Furthermore, as the SNP coverage and sample size of eQTL datasets available in brain tissue increases, this will allow for better instrumentation of existing genes and for more genes to instrumented, improving the scope of MR analysis (to include trans-effects for example) that can be carried out and potential to discover further loci. Other brain QTLs (such as methylation, splicing, protein and cell-type QTLs) can also provide valuable information on underlying biology, therefore applying a multi-omic approach to future target identification efforts will be a highly fruitful avenue of MR research.

## Materials and methods

### Primary MR analysis: Two sample MR between brain eQTLs and neurological and psychiatric disease traits

#### Primary outcome selection

We selected 12 traits that were either neurological or psychiatric conditions as the primary outcomes (S14 Table) for this study. The 7 psychiatric traits included: anorexia nervosa [44], attention deficient disorder [45], autism spectrum disorder [46], bipolar disorder [47], major depressive disorder [48], obsessive compulsive disorder [49], and schizophrenia [50]. The 5 neurological traits included: Alzheimer’s disease, amyotrophic lateral sclerosis [51], frontotemporal dementia [52], multiple sclerosis [53], and Parkinson’s disease.

For Alzheimer’s disease, we combined the summary statistics from the Lambert et al GWAS [54] (17008 cases, 37154 controls) with a GWAS by proxy of Alzheimer’s disease family history in the UK Biobank (56773 proxy-cases and 379370 controls). Similarly, for Parkinson’s disease, we combined summary statistics from Nalls et al GWAS [37] (13708 cases, 95282 controls) and a GWAS by proxy of Parkinson’s disease family history in the UK Biobank (16909 proxy-cases, 388689 controls). For the UK Biobank analyses, we defined proxy-cases as participants who answered yes to whether they have a biological father (UK Biobank field 20107), mother (UK Biobank field 20110) or sibling (UK Biobank field 20111) who suffered from Alzheimer’s disease/dementia (or Parkinson’s disease). Participants who answered “Do not know” or “Do not with to answer” were excluded from analyses. All other individuals were included as controls. We further excluded participants of non-European ancestry [55]. Genome-wide association analyses were performed using BOLT-LMM [56]. To enable meta-analysis with the previous GWAS, we multiplied the log(OR)-scale effect sizes and standard errors by a factor of two [57], and then performed meta-analysis using a fixed-effects inverse-variance weighted approach.

#### Instrument selection and preparation

We obtained our genetic instruments from a cis-eQTL meta-analysis performed on cohorts from AMP-CMC. This dataset consisted of a total of 6,937,060 SNPs across 19,281 gene transcripts measured in 1,286 pre-frontal brain cortex tissues. We removed 66,788 SNPs within the Major Histocompatibility (MHC) region (defined as SNPs between 24Mb and 36Mb in chromosome 6 hg19 build) from the dataset due to the extensive LD structure and complexity of this region. We then selected 1,054,129 genome-wide significant SNPs using P<5x10^-8^ as a threshold and conducted LD clumping on this set of SNPs to obtain a set of independent eQTL instruments for each gene transcript. A total of 7,689 SNPs across 7,137 genes remained as instruments after clumping. LD clumping was performed using the clump_data function provided by the TwoSampleMR R package [43] on the default settings where all SNPs present in the 1000 Genomes EUR population with r^2^>0.001 within a 10Mb window of the top hit were removed.

We standardised all eQTL SNP effects (*β_j_*~*N*(0,1)) to represent a per SD change in gene expression to allow for comparison of the magnitude of the MR effect sizes across all genes for the different diseases. Standardised effect sizes and standard errors (SE) were derived using the following formulae [9,43]:

$$\beta_{standarised}=\frac{Z_{j}}{\sqrt{2*{MAF}_{j}(1-{MAF}_{j})*(N_{j}+Z_{j}^{2})}}$$

$${SE}_{standarised}=\frac{1}{\sqrt{2*{MAF}_{j}(1-{MAF}_{j})*(N_{j}+Z_{j}^{2})}}$$

where the z score Z_j_ is obtained for SNP_j_ by dividing the raw SNP effect by the SE of the effect, and the variance of SNP_j_ genotypes is derived from the study’s allele frequencies using 2*MAF_j_(1-MAF_j_), N_j_ is the sample size for the SNP (reported sample size for the study was used if SNP-specific sample size was not available). To determine instrument strength we calculated the F-statistic for each instrument using the Cragg-Donald statistic [58,59]:

$$PVE=\beta^{2}/(\beta^{2}+{se}^{2}*n)$$

where PVE is the proportion of variance explained by the eQTL instrument, *β* and se is the effect size and standard error of the instrument, n is the sample size of the instrument (n = 1286 for our brain eQTL).

Fstatisitc=PVE*(n−1−k)/(1−PVE*k)

where k is the number of instruments used in the MR estimate (k = 1 for single SNP MR) and n is the instrument sample size if k = 1 or average of the sample size over k instruments if k>1.

#### Data harmonisation and two sample MR analysis

We extracted the selected instruments from the outcome GWASs, harmonised the genetic association between the instrument and outcome GWAS so that they reflected the same allele, and then conducted MR analyses on the harmonised data. We estimated the effect of gene expression on the primary outcomes using a single SNP instrument (Wald ratio method). All these steps were implemented using the TwoSampleMR R package [43] version 0.4 maintained by MR-Base.

#### Selection of top MR findings

A Bonferroni correction threshold of P = 6x10^-7^ at *α* = 0.05 based on n = 80,557 MR tests was used to prioritise gene-outcome associations for further follow up. Some of the prioritised genes were within the same genomic region. For these cis-genes, in order to visualise the LD between instruments, regional association plots were generated in LocusZoom (locuszoom.org) [60] using 1000 Genomes EUR reference panel to compute the LD relative to the Wald ratio estimate for the gene with the lowest p-value in the region. LocusZoom plots are available for viewing on GitHub repository (denisbrd/braineQTLMR_datasets; DOI: 10.5281/zenodo.3778433).

### Colocalization analysis between gene expression and traits

Colocalization analysis was conducted on the identified gene-outcome MR associations to confirm that the gene expression exposure and outcome shared the same causal variant within the region of interest (rather than the variant being shared coincidentally due to correlation through LD). Colocalization compares the probability of five hypotheses across a region: neither gene expression or the outcome are associated with genetic variants in the region (H_0_), only gene expression is associated with a genetic variant in the region (H_1_), only the outcome is associated with a genetic variant in the region (H_2_), gene expression and outcome are both associated with the region, but with different causal variants (H_3_), gene expression and outcome are associated with the same causal variant (H_4_).

We extracted the summary association statistics for all SNPs residing within a 1Mb region surrounding the instrument (i.e. instrument +- 500kb) from the eQTL dataset, and then extracted the summary association statistics for the same SNPs from the outcome GWAS. We then used Bayesian coloc.abf function in R to conduct colocalization analyses on the SNPs common to both datasets [41]. Default priors were used: the expected proportion of SNPs associated with gene expression (p_1_ = 5x10^-4^), associated with the outcome (p_2_ = 5x10^-4^) and associated with both gene expression and the outcome (p_3_ = 5x10^-5^). We considered the traits to colocalize if the posterior probability (PP_4_) for H_4_ was greater than 70%.

### Instrument comparison between our study and other eQTL datasets

In order to determine the extent of similarity which would be observed from conducting the same MR analysis in other eQTL datasets, we compared the gene expression effect estimates for our instruments (from the AMP-CMC brain eQTL dataset) to the effect estimates for the same SNP in other eQTL datasets. In order to compare against brain tissue, we downloaded the full summary association statistics for the two brain eQTL dataset used in Qi publication [10] from their SMR website (https://cnsgenomics.com/software/smr/#DataResource): AMP, CMC and GTEx meta-analysis on brain cortex tissue (n = 1194) and the meta-analysis across ten different brain tissues available in GTEx (n = 233). In order to compare against blood tissue, we acquired the full summary statistics for eQTLGen [16] (n = 31,684) from their website (https://www.eqtlgen.org). In order to conduct a cross-tissue comparison, we downloaded the full association summary statistics available across all 48 tissues in GTEx version 7 (https://gtexportal.org/home/) [61]. We then extracted our instruments from each of these eQTL datasets, matching by both dbSNP rsid for the SNP and Ensembl ENSG gene identifier. We calculated the Pearson’s correlation between the eQTL effect sizes in our study and the other studies across the instruments. We also determined the number of times our instruments had a gene expression effect which was significant at P<5x10^-8^ (cut-off used for instruments in our study) and P<0.05 level in the other eQTL datasets as well as the number of times our instruments disagreed with the direction of gene expression effect in other datasets (provided P<0.05 for the gene expression effect in the other dataset).

### Follow up MR analysis on selected instruments

The following analyses were conducted on the selected instruments identified in the main MR analysis (i.e. for the potentially causal genes which passed the Bonferroni correction).

#### Assessment of pleiotropy phenome-wide

We conducted a MR phenome-wide association analysis (MR-PheWAS) on 219 disease outcomes and risk factors, previously collated by Zheng et al [62] (S15 Table) to assess the potential for pleiotropic relationships across traits. Colocalization was performed on the MR effects which had P<0.05

Using the MR-PheWAS dataset, we also examined whether the proportion of Wald ratio effects that had a shared effect (P<0.05) in our primary MR could have been through random chance alone. We did this through permutation testing, in which we generated a distribution based on repeatedly randomly selecting (n = 10,000 iterations) the same number of non-brain related traits (n = 12 selected from 182 in total) from the MR-PheWAS dataset. In each iteration, we determined the number and proportion of effects which reached P<0.05. From this distribution, we could then estimate the 95% empirical confidence interval around the proportion and p-value (number of times each iteration had a proportion that as least as extreme as our test statistic (proportion = 141/551 = 0.26). We checked the distribution and it was approximately Normal: descriptive parameters were as follows: number of effects at P < 0.05: min = 17, mean = 61.9, max = 140, proportion of effects at P<0.05, min = 0.037, mean = 0.12, max = 0.27, empirical 95% CI for proportion = 0.12, [0.07,0.17].

#### Assessment of molecular pleiotropy

We assessed the number of different genes whose expression was associated with each of our instruments (i.e. molecular pleiotropy). We retrieved the summary association statistics across the gene transcripts available in the AMP-CMC study and determined the genes which were associated with the instrument at Bonferroni P-value *α* = 0.05. We then conducted colocalization analysis between the gene expression and the outcome detected in our primary MR.

#### Assessment of tissue-specific effects

We assessed consistency between our selected instruments and brain tissue eQTLs from the Genotype-Tissue Expression (GTEx) project version 7 [61]. The 13 brain tissues available in GTEx were examined: Amygdala (n = 88), Anterior cingulate cortex BA24 (n = 109), Caudate basal ganglia (n = 144), Cerebellar Hemisphere (n = 125), Cerebellum (n = 154), Cortex (n = 136), Frontal Cortex BA9 (n = 109), Hippocampus (n = 111), Hypothalamus (n = 108), Nucleus accumbens basal ganglia (n = 130), Putaman basal gangli (n = 111), Spinal cord cervical c-1 (n = 83), and the Substantia nigra (n = 80). We extracted our selected instruments from these datasets, and we determined the number of GTEx eQTLs across the brain tissues that showed evidence of association (using a Bonferroni corrected *α* = 0.05) and had the same direction of effect as our instruments (from AMP-CMC study). We also computed the Pearson’s pairwise correlation between our gene expression effects and GTEx effects in each tissue. In order to assess whether eQTL effects showed statistical evidence of varying across different brain tissues, a meta-analysis was performed across the 13 brain GTEx tissues using the GWAMA meta-analysis package [63]. We defined evidence for heterogeneity across tissues as P<0.001 based on Cochran’s Q test. We also examined if using our selected instruments in blood tissue could proxy for brain tissue effects in an MR analysis. To do this summary association statistics were retrieved from eQTLGen (https://www.eqtlgen.org) [16]. We performed colocalization analysis between the gene expression effects measured in brain (AMP-CMC) and blood (eQTLGen) and for the regions which passed colocalization analysis (PP_4_>70%), we determined the number of blood effects which were genome-wide significant (P< 5x10^-8^) and had the same direction of effect as the brain instrument. We also computed the Pearson’s pairwise correlation between brain and blood effects across our selected instruments.

#### Assessment of directionality of MR effect

A potential issue with the MR analysis could be that the gene expression changes are a consequence of genetic predisposition to disease rather than a cause of the disease. Directionality can be determined using either bi-directional MR [64] or Steiger filtering [65,66]. For a bi-directional MR, a reverse MR is conducted where the SNPs associated with the outcome are used as instruments, therefore evidence of a relationship would imply an incorrect direction of effect from the outcome to the exposure. The Steiger filtering method compares the effect estimates for the instrument-exposure association (*ρ_gx_*) and instrument-outcome association (*ρ_gy_*) in order to infer the direction of causality. The Steiger test computes a p-value (p_steiger_) which is the probability of obtaining a difference between these two correlations (*ρ_gx_*−*ρ_gy_*) at least as extreme as the one observed in the study under the null hypothesis. The p_steiger_ statistic indicates the strength of evidence that the relationship is in the correct direction from exposure to outcome. Since we did not have access to full summary association statistics for in the AMP-CMC study (i.e. only captured cis-acting regions), it was not possible to conduct bi-directional MR in our brain tissue dataset, but we could perform the Steiger filtering test. Steiger filtering was conducted on our instruments using the mr_steiger function in the TwoSampleMR package, and those labelled as not having passed the mr_steiger filter reported if present. We performed bi-directional MR using eQTLGen as the outcome dataset in the reverse MR. For the reverse MR step, we first defined instruments for outcomes by selecting genome-wide significant (P<5x10^-8^) SNPs from our primary neurological and psychiatric GWASs, and performed LD clumping on the selected SNPs. We then looked up these SNPs in the genes available within eQTLGen, harmonised the effects and performed MR analyses to assess the effect of the neurological and psychiatric traits on gene expression using IVW linear regression, MR Egger and the weighted median estimator via the TwoSampleMR R package.

### Evaluation of existing knowledge to assess potential drug targets

We carried out lookups in two public databases to assess the functional relevance and potential druggability of the genes identified in the main MR analysis: (i) Online Mendelian Inheritance in Man (OMIM) [67] to search for human monogenic disorders relating to the gene. Gene names were searched for in Online Mendelian Inheritance in Man (https://omim.org) online and the allelic variants section was manually reviewed for evidence of mutations which caused abnormal neurological or behavioural phenotypes and (ii) Open Targets [68] to find potential drug target-indication pairs for the gene. Ensembl ENSG gene name was searched in the Open Targets Genetics database online (https://genetics.opentargets.org) and clinical trials information was collected for genes which were annotated as being potentially druggable, which we defined as the target having entered the clinical trial stage and having at least completed Phase 0.

## Supporting information

S1 Fig Flow chart providing overview of instrument selection, MR analysis and drug target prioritization steps used in this study. Wald ratio (WR) and coloc prob (posterior probability of colocalization). Outcome GWAS datasets used in the MR analysis are reported in oval boxes and main findings reported in square boxes.
(PDF)

S1 Table Summary association statistics comparison for instruments from our study (AMP-CMC) which had a different direction of gene expression effect to other eQTL datasets (Qi brain eQTL studies and across GTEx version 7 tissues). (XLSX)

S2 Table Correlation analysis results between gene expression effects for our instruments (AMP-CMC) and different tissue types present in GTEx version 7. (XLSX)

S3 Table Descriptive summary of the number of GWAS associations, instrumented genes and top MR findings across our primary outcomes. (XLSX)

S4 Table Harmonised SNP effects, Wald ratio statistics and colocalization results for the genes detected in the main MR analysis. (XLSX)

S5 Table MR and colocalization results for the prioritised genes found to be related with other non-brain related traits in our MR-PheWAS. (XLSX)

S6 Table eQTL effects and colocalization results for our prioritised gene instruments found to be associated with multiple cis-genes in the genomic region. Gene identified in the main MR analysis is highlighted in bold. Colocalization was also performed between the other cis-gene(s) and the outcome identified in the main MR analysis for our prioritised gene.
(XLSX)

S7 Table Number of different tissues in GTEx version 7 our selected instruments shared gene expression (eQTL) association with. Number of tissues in which gene expression effect was available in GTEx (tssue.count) for our instrument, number of tissues where the gene expression effect passed the Bonferroni correction threshold (P<9x10-5), flag to indicate whether our instrument association was reproduced in GTEx tissue which we defined as Bonferroni significant tissue associations which had the same direction effect as our instrument (reproduce.tissue).
(XLSX)

S8 Table Heterogeneity results from the meta-analysis of eQTL effects across the 13 brain tissues available in GTEx version 7. Analysis was conducted on our prioritised gene instruments.
(XLSX)

S9 Table Summary association statistics from our comparison between our prioritised gene instruments and blood eQTLs from eQTLGen. (XLSX)

S10 Table Comparison between instruments derived in eQTLGen and AMP+CMC studies. AMPAD.shared (0/1 flag if gene is also instrumented in AMP+AD + CMC MR analysis) and AMP.AD.tophit.instrument (rsid of instrument used in AMP_AD + CMC, if multiple instruments available for the gene then rsid with the lowest p-value is reported).
(XLSX)

S11 Table MR results from our reverse MR conducted between primary outcomes and gene expression in eQTLGen. (XLSX)

S12 Table Steiger filtering analysis results conducted on our prioritised gene instruments. (XLSX)

S13 Table Descriptive table detailing the findings from our drug target evidence follow up on our prioritised genes. (XLSX)

S14 Table Descriptive statistics of the studies which were selected as the primary outcomes for our MR analysis. (XLSX)

S15 Table Descriptive statistics for the risk factors and disease outcomes tested in our MR-PheWas. (XLSX)
