# Supplementary material for: Identifying drug targets for neurological and psychiatric disease via genetics and the brain transcriptome
Source: PLoS Genet. 2021 Jan 8;17(1):e1009224. doi: 10.1371/journal.pgen.1009224 (PMC7819609; doi:10.1371/journal.pgen.1009224)
Supplement: S1 Fig — Wald ratio (WR) and coloc prob (posterior probability of colocalization). Outcome GWAS datasets used in the MR analysis are reported in oval boxes and main findings reported in square boxes. (PDF) [file pgen.1009224.s001.pdf]

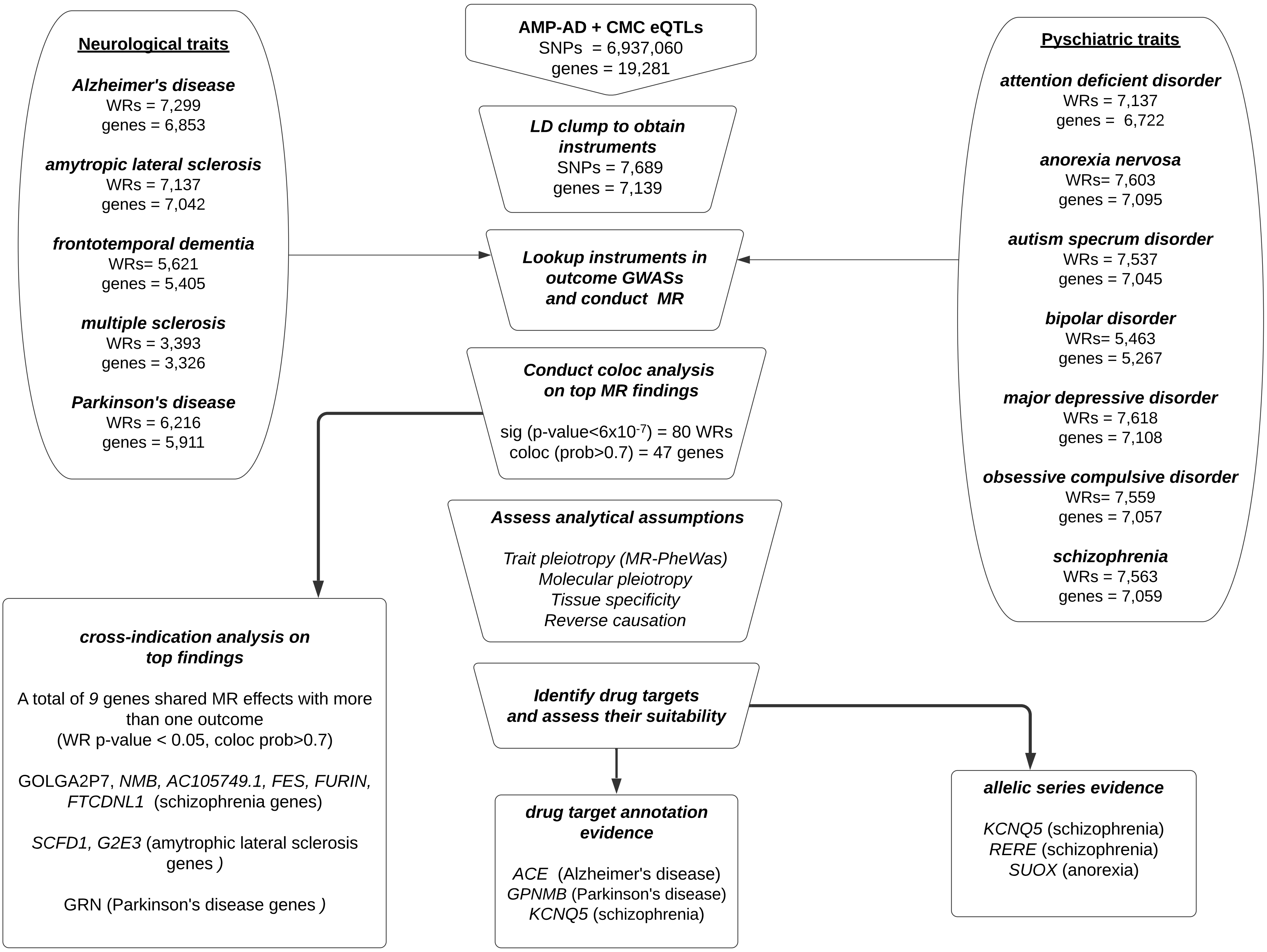
Neurological traits
Alzheimer's disease
WRs = 7,299
genes = 6,853
amytropic lateral sclerosis
WRs = 7,137
genes = 7,042
frontotemporal dementia
WRs= 5,621
genes = 5,405
multiple sclerosis
WRs = 3,393
genes = 3,326
Parkinson's disease
WRs = 6,216
genes = 5,911
AMP-AD + CMC eQTLs
SNPs = 6,937,060
genes = 19,281
LD clump to obtain instruments
SNPs = 7,689
genes = 7,139
Lookup instruments in outcome GWASs and conduct MR
Conduct coloc analysis on top MR findings
sig (p-value<6x10^{-7}) = 80 WRs
coloc (prob>0.7) = 47 genes
Assess analytical assumptions
Trait pleiotropy (MR-PheWas)
Molecular pleiotropy
Tissue specificity
Reverse causation
Identify drug targets and assess their suitability
Pyschiatric traits
attention deficient disorder
WRs = 7,137
genes = 6,722
anorexia nervosa
WRs= 7,603
genes = 7,095
autism specrum disorder
WRs = 7,537
genes = 7,045
bipolar disorder
WRs= 5,463
genes = 5,267
major depressive disorder
WRs = 7,618
genes = 7,108
obsessive compulsive disorder
WRs= 7,559
genes = 7,057
schizophrenia
WRs = 7,563
genes = 7,059
cross-indication analysis on top findings
A total of 9 genes shared MR effects with more than one outcome
(WR p-value < 0.05, coloc prob>0.7)
GOLGA2P7, NMB, AC105749.1, FES, FURIN, FTCDNL1 (schizophrenia genes)
SCFD1, G2E3 (amytrophic lateral sclerosis genes)
GRN (Parkinson's disease genes)
drug target annotation evidence
ACE (Alzheimer's disease)
GPNMB (Parkinson's disease)
KCNQ5 (schizophrenia)
allelic series evidence
KCNQ5 (schizophrenia)
RERE (schizophrenia)
SUOX (anorexia)
